# Environmental DNA detection of giant snakehead in Thailand’s major rivers for wild stock assessment

**DOI:** 10.1371/journal.pone.0267667

**Published:** 2022-05-10

**Authors:** Maslin Osathanunkul, Panagiotis Madesis

**Affiliations:** 1 Department of Biology, Faculty of Science, Chiang Mai University, Chiang Mai, Thailand; 2 Research Center in Bioresources for Agriculture, Industry and Medicine, Chiang Mai University, Chiang Mai, Thailand; 3 Institute of Applied Biosciences, Centre for Research & Technology Hellas (CERTH), Thessaloniki, Greece; 4 Laboratory of Molecular Biology of Plants, Department of Agriculture, Crop Production and Rural Environment, University of Thessaly, Volos, Magnesia, Greece; University of Hyogo, JAPAN

## Abstract

Capture-based aquaculture is now gaining much attention in Southeast Asia. This system was used to produce several fish species with social and economic implications, including the giant snakehead (*Channa micropeltes*). As wild harvesting of organisms for seed stock is one of main practices in capture-based aquaculture, abundance and distribution of the wild stock are essential for both environmental impact evaluation and stock management. Mark and recapture, visual observation and physical capture of target species are costly, ineffective, and labour intensive for fish surveys in several cases. Detection of target organisms using eDNA (environmental DNA) could be a good alternative as it has proved to be a non-invasive, rapid, and sensitive method for aquatic species monitoring and surveying. Here, we developed a TaqMan assay that targets the 16S region of giant snakehead DNA to amplify eDNA captured in water samples. 300 µl of water samples were collected from 15 sites located in the Chao Phraya River Basin (Ping, Wang, Yom, Nan, and Chao Phraya River) and filtered with 0.7 µm glass fibre membrane filter. Giant snakehead eDNA was detected in most tributaries (60%) with concentrations ranging from 74.0 copies/ml in Wang River sites to 7.4 copies/ml in Nan River sites. As intensification of capture-based aquaculture could lead to depleting of wild fish stocks, urgent management is needed. However, the existing conventional approaches for assessment of fish overexploitation, survey and monitoring have several limitations.

## Introduction

Freshwater aquaculture has been long practiced. Aquaculture offers many advantages for people providing nutritious, protein-rich food, and income. In 2018, aquaculture production was reported to be 82.1 million tonnes (250 billion USD) which 51.3 million tonnes are from inland aquaculture (FAO, 2020). Consumption of food fish is increasing with average annual rate of 3.1 percent from 1961 to 2017. Fish is a primary source of high-quality protein which accounted for 17% of the animal protein consumed worldwide [1].

Capture-based aquaculture is one of many common practices in the river basin throughout the world. In capture-based aquaculture, fish are caught from rivers, streams, lakes, reservoirs, ponds, channels, paddy-fields, etc. Species are captured from the wild (usually very early stages in the life cycle) stocked into confined and controlled conditions where they are raised to a marketable size. Some capture-based aquaculture industries employ a mix of hatchery-produced and wild-caught aquatic animals for subsequent use in aquaculture [2]. However, hatcheries and nurseries are costly and require infrastructure to manage. Therefore, use of wild-caught seedstock is popular in many remote areas. While aquaculture production has grown rapidly, wild fish stocks have become significantly depleted. Intensification of aquaculture could lead to negative impacts on environments. The Chao Phraya River is among the most productive inland fisheries in the world [3]. One group of interesting fish taxa is the snakeheads (Channidae) with two of these currently used in significant numbers for aquaculture: the giant snakehead (*Channa micropeltes*) and the Chevron snakehead (*Channa striata*). In Thailand, the giant snakehead is one of the top 20 species with economic importance which is caught from inland waters [4]. Both adult and juvenile fisheries of the giant snakehead are common in the Songkhram River (Thailand) and the Mekong River (Cambodia, Lao People’s Democratic Republic, and Viet Nam). The giant snakehead is native to Southeast Asia, where it occurs in Indonesia, Laos, Malaysia, Thailand, and Vietnam [5]. However, quantitative data about wild giant snakehead seed capture is currently limited. There is no report of such information in Thailand and Lao People’s Democratic Republic, and only a few reports in Cambodia and Viet Nam [6].

In Thailand, the Chao Phraya River is formed by four major tributaries: The Ping, Wang, Yom, and Nan Rivers, which flow from the northern watershed of Thailand to merge in the middle of country. The Chao Phraya Basin, the largest river basin in Thailand, covers around one third of Thailand’s territory which includes the great majority of irrigated areas and the Bangkok Metropolitan Area [7]. It is suspected that the giant snakehead inhabits all the rivers in the basin. Recently, there was only a fish survey in the Nan River basin (The Nan River and its eight tributaries) in which giant snakehead were caught in the Nan River and one of the tributaries (Ha River) [8]. Thus, an increased understanding regarding the abundance and distribution of the giant snakehead in Thailand, particular the Chao Phraya Basin, would be useful.

As mentioned earlier, giant snakehead caught from the wild and then are raised in water cages as food is popular in many areas of Asia including Thailand. In addition, the giant snakehead was introduced into several countries such as China, the Philippines, Singapore, and the United States. The fish is one of the major threats to the native fish fauna there as it is a large predatory fish [5, 9, 10]. Also, the giant snakehead and other snakehead species are common aquarium trade species and could be found in pet shops in most EU countries [11]. Spreading of snakehead into EU waters is now a matter of concern [5]. Therefore, eDNA monitoring or detecting the giant snakehead in the wild could be useful not only for Thailand but other several countries as well.

Fish surveys in such a large river basin are difficult and require much logistical effort (e.g., time, money, and labour). There are several conventional fish survey methods that include the use of fishing gears, electrofishing, and visual surveys. The main limitations of these traditional survey are their varying efficiency, incomplete sampling, and labour-intensiveness [12, 13]. The eDNA-based method has been pointed out as more sensitive [14–22] and cost less [23–25] than physical capture or visual observation. Recently, the use of environmental DNA (eDNA), defined as short DNA fragments that an organism sheds into their native environment, was demonstrated to be a powerful tool for monitoring biodiversity in near real-time [e.g., 26–28]. Detection of the giant snakehead in the Chao Phraya River Basin via eDNA, instead of the traditional direct observation method, could be more sensitive and less time-consuming. In addition, eDNA monitoring of the giant snakehead could benefit capture-based aquaculture in countries where it is a common practice such as Cambodia, Malaysia, Thailand, and Vietnam.

## Materials and methods

### Ethics statement

This study was approved by the Animal Care and Use Committee review (The Laboratory Animal Center, Chiang Mai University) under protocol number: 2561/FA-0001.

### Sampling sites

**Fig 1:**
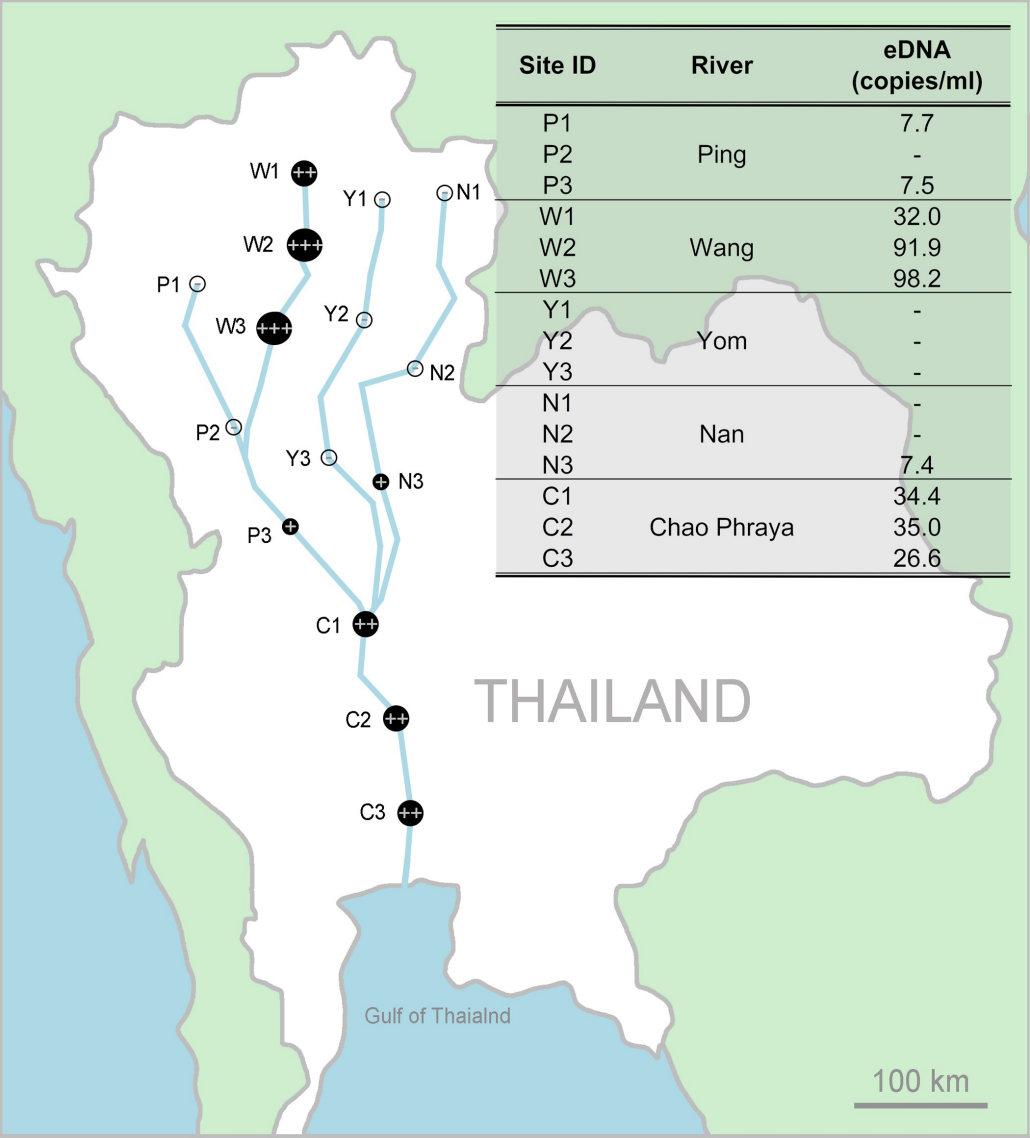
Map showing sampling sites in the Chao Phraya River Basin. Sites on the Ping River (P1-P3), sites on the Wang River (W1-W3), sites on the Yom River (Y1-Y3), sites on the Nan River (N1-N3) and sites on the Chao Phraya River (C1-C3). Giant snakehead eDNA concentration (copies per ml) detected at each site is shown.

Water samples were collected from sites within the Chao Phraya River Basin. Three sites were sampled in the Chao Phraya, Ping, Wang, Yom, and Nan Rivers (n = 15 sites total) in January–February 2018 during the middle of the dry season (Fig 1). Of note is that the Ping, Wang, Yom, and Nan Rivers are the main tributaries of the larger Chao Phraya River. To avoid contamination, all field equipment was sterilized using 10% bleach, UV-Crosslinker or autoclaved and sealed prior to transport to the study site, and a separate pair of nitrile disposable gloves were used to collect each sample. At each site, three replicate water samples were collected from a bucket that had been previously decontaminated with a 10% bleach rinse followed by two distilled water rinses. Each site was sampled in triplicate by collecting 300 ml water samples and immediately filtered on 0.7 μm pore size filter (Whatman International Ltd., Maidstone, UK) in the field using a handheld vacuum pump (Thermo Fisher Scientific Inc., MA, USA). For every sampling day, deionised water (300 ml) was filtered in the field as a negative control.

### qPCR assay

All the DNA analysed originated from the mucus of five individuals of giant snakehead. Total DNA was extracted from the mucus sample using the Qiagen Dneasy Blood and Tissue Kit (Qiagen, Valencia, CA). Extracted DNA was used as a template for qPCR assay together with 127 bp long synthetic fragment which is a replica of giant snakehead 16S rRNA sequence (Integrated DNA Technologies Pte. Ltd., Singapore). DNA samples were quantified using a Qubit fluorometer (Life Technologies) calibrated with the Quant-iT dsDNA HS Assay following the manufacturer’s instructions. For each replicate, 3 µl volumes were measured. The mitochondrial 16s rRNA fragments (127 bp) were amplified and quantified using the following primers and TaqMan probe from Osathanunkul and Minamoto, 2021 [29]: Cmi-F -Forward: 5′- CTCGCACCAACTAGGCTTTTCC -3′, Cmi-R -Reverse: 5′- GGTTAATGTTCGGTGGATTGTCCGT -3′, and Cmi-PR -Probe: 5′-FAM- TGCTAACATGGAAGCACTTA -MGB-3′. Species specificity of the primers and probe was confirmed by Osathanunkul and Minamoto, 2021 [29] through *in silico* and *in vitro* checking with closely related species (species in the same genus: *Channa marulius* and *Channa striata*) and non-target species (*Barbonymus altus*, *Chitala ornate*, *Probarbus jullieni*, and *Puntioplites proctozysron*) that potentially inhabiting the sampling area. In addition, five species co-occur in this river system including *Barbonymus gonionotus*, *Cyprinus carpio*, *Pangasianodon hypophthalmus*, *Pangasius bocourti*, and *Pangasius larnaudii* were tested.

A positive-control water sample was collected from Kwan Phayao Lake wherein giant snakehead is known to inhabit [30]. All eDNA qPCR amplifications were performed in three replicates in a final volume of 20 µl, using 10.0 µl of 2× TaqMan Environmental Master Mix 2.0 (Thermo Fisher Scientific), 2.0 µl of DNA template, 900 nM each of the F/R primers, and 125 nM of the probe. Samples were run under the following conditions: an initial 10 min incubation at 95°C followed by 50 cycles of denaturation at 95°C for 15 s and annealing/extension at 60°C for 1 min. Both lab and field negative controls with all PCR reagents but no template (three replicates) were run in parallel to assess potential contamination. The quantification cycle (Cq) was converted to quantities per unit volume using the linear regression obtained from the synthesized target 16S rRNA gene standard curve. The giant snakehead eDNA concentrations were then reported as copies/ml. The limit of detection (LOD) and the limit of quantification (LOQ) were also measured using the standard dilution series of synthesised target gene fragments with known copy numbers. A dilution series containing 1.5 × 10^1^ to 1.5 × 10^4^ copies per reaction were prepared and used as quantification standards. The calculation of LOD and LOQ was done using R script published by Klymus et al. 2020 [31].

### DNA extraction from the filters

Filters were placed in individual 1.5 ml tubes and kept in a polystyrene box containing dry ice before transferring to a − 20°C freezer until extraction. The filters were extracted within 48 hr of sampling using Qiagen Dneasy Blood and Tissue Kit (Qiagen, Hilden, Germany) using a protocol modified from the manufacturer’s protocol with the following changes: the DNA from all samples was eluted twice with 25 µl AE buffer, in a total volume of 50 µl to obtain a more concentrated eDNA solution. The doubled volume of ATL buffer (360 µl), Proteinase K (40 µl), AL buffer (400 µl) and Ethanol (400 µl) were used. Each eDNA samples was then treated with the OneStep PCR Inhibitor Removal Kit (Zymo Research) to remove potential PCR inhibitors bound to eDNA molecules that may be present in water samples before using in qPCR assay.

## Results

The species-specific TaqMan qPCR assay showed that only DNA from the giant snakehead mucus amplified, not in the negative controls. The TaqMan assay standard curve exhibited the following characteristics: slope = −3.404, Y inter = 38.007, R^2^ = 0.991, Eff% = 96.703. The LOD was 8.90 copies per reaction and the LOQ was 9.10 copies per reaction. Giant snakehead eDNA was detected at one site each on the Ping and Nan Rivers (Fig 1; sites P3 and N3). Giant snakehead eDNA was found at all sites on the Wang and Chao Phraya rivers. On the other hand, no giant snakehead eDNA was detected at any sites on the Yom River. Where detected, concentration of giant snakehead eDNA varied from 7.4–98.2 copies per ml (Fig 1). The highest concentration of giant snakehead eDNA was found at site W3 (98.2 copies/ml) and the lowest was at site N3 (7.4 copies/ml). The average concentration of giant snakehead eDNA per sampled river was as follows: 7.6 copies/ml (Ping River), 74.0 copies/ml (Wang River), 0 copies/ml (Yom River), 7.4 copies/ml (Nan River) and 32.0 copies/ml (Chao Phraya River).

## Discussions

The Food and Agriculture Organization of the United Nations estimates that capture-based aquaculture contributes about 20 percent of all the aquaculture production [1]. It is not a small number and thus should receive more attention in several aspects such as environmental impact, production economics, and resource management. Because of capture-based aquaculture’s linkage to capture fisheries, stock overexploitation is one of the major concerns. Overfishing of some seed species in the wild has already been reported, e.g., grouper, milkfish, and mud crab [32]. Movement, migration, and natural mortality data are normally required for stock assessment with the mark and recapture method used to gain such data.

The Chao Phraya River Basin is one of the most important basins not only in Thailand but Southeast Asia. The abundance of the giant snakehead has not been recorded in the studied river basin despite the social and economic value of the species in the region. Giant snakehead eDNA was detected in the lake where they are reported to be found and in the downstream Chao Phraya, Ping, Wang, and Nan Rivers with varying concentrations (Fig 1). No eDNA was amplified from water collected at the sampling sites on the Yom River (Y1-Y3). Although, there was no reliable statistics of the distribution of giant snakehead in the Chao Phraya River Basin. The giant snakehead catches were reported by local fishermen in the Chao Phraya River and all four tributaries. Therefore, eDNA of giant snakehead were expected to be found even in the Yom River. The species-specific TaqMan assay presented here only amplify DNA from the giant snakehead and did not amplify DNA from any of the non-target species tested. The amplified fragments from positive qPCR assay were also randomly chose and sent out for sequencing to confirm that positive detections were true positives (data not shown). However, two main things to be considered why eDNA have not been detected in the Yom River are that filter pore size and sampled volume. Recommended pore sizes in the literature range from 0.2 µm to 1.2 µm [33–36]. Several studies indicated that small pore size filters (≤0.45 µm) could yield the most eDNA but limit water volume and speed of filtration [37, 38]. However, they clog easily in turbid waters so that there could be a trade-off between the amount of water that can be filtered and the filter pore size [20, 35, 37, 39]. In eDNA detecting fish species studies, 1 or 2 L water collection and eDNA capture on 0.7 μm filters were most used [40]. However, it was hard to filter such volume of water in this study because of filter clogging. The eDNA capture was found to be very sensitive to the volume of water filtered, as previously reported [41]. Use of too small water volumes (15–75 ml) could lead to failure in quantifying fish populations [42]. If possible, larger water volumes should be collected to ensure reliable detection rate of studied species [43–44]. Nevertheless, selection of the appropriate filter is dependent on the water sample. Here, 300 ml water samples were the maximum volume which could be filtered on 0.7 μm pore size filter.

## Conclusions

The continued growth of aquaculture production is crucial as the world heads towards an increasing world’s population. Intensification of capture-based aquaculture could lead to depleting of wild fish stocks. Thus, urgent management is needed because the existing conventional approaches for assessment of fish overexploitation, survey and monitoring have several limitations. The eDNA-based method has been shown to be an effective alternative method for fish detection or monitoring, which could greatly advance assessment and enable understanding of the threats to the ecosystems. The method will be useful in planning sustainable resource management. In addition, it would be useful not only for evaluation of native distribution throughout major river systems but also for monitoring rivers for aquaculture farm escapees.

## Supporting information

S1 Graphical abstract (DOCX)
