# Supplementary figures and images for: Environmental DNA detection of giant snakehead in Thailand’s major rivers for wild stock assessment
Source: PLoS One. 2022 May 10;17(5):e0267667. doi: 10.1371/journal.pone.0267667 (PMC9089910; doi:10.1371/journal.pone.0267667)

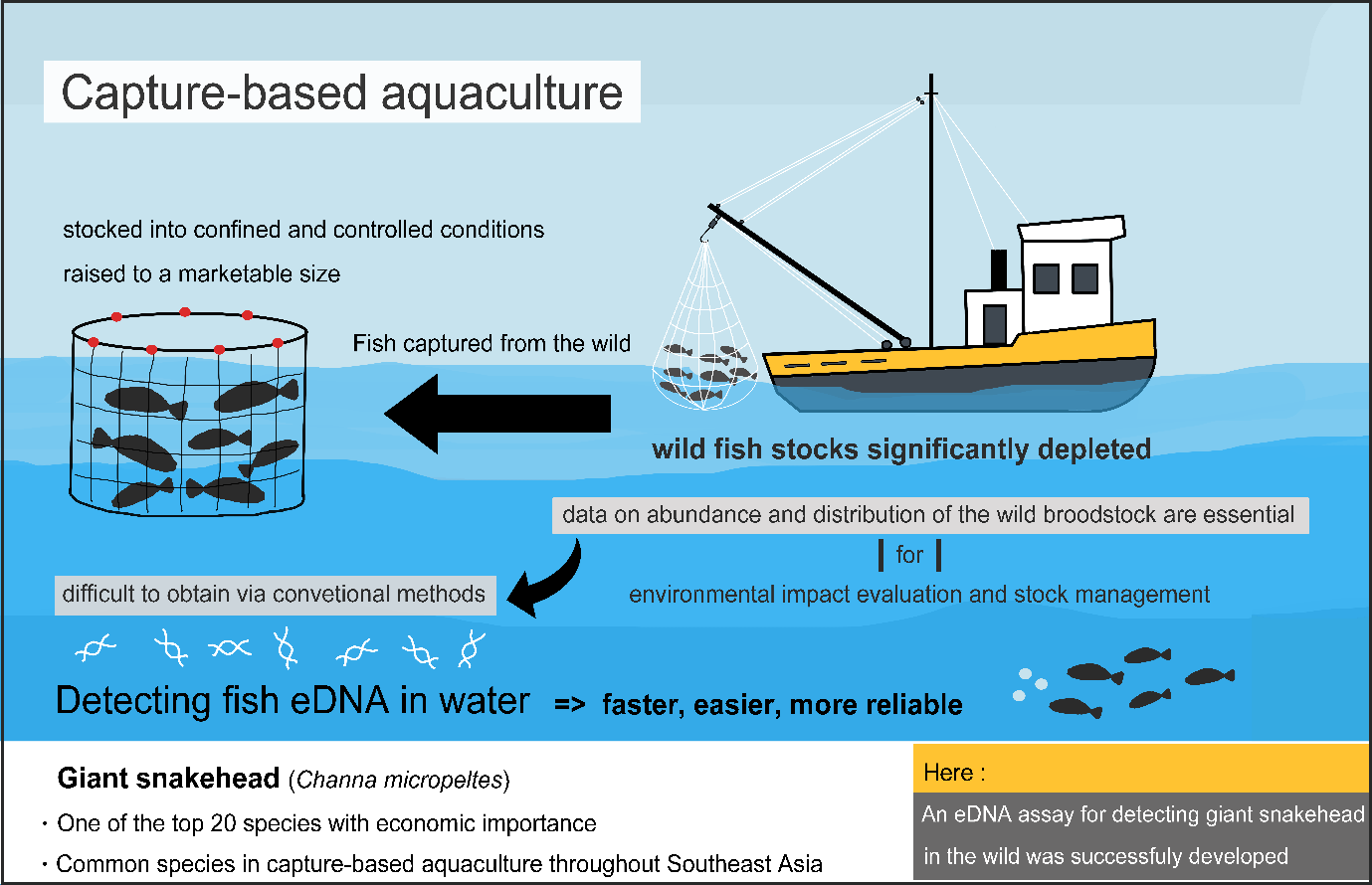

Supplement: S1 Graphical abstract — (DOCX) [file pone.0267667.s001.docx]
